# From Ethnobotany to Pharmacognosy: A Review of *Firmiana colorata*

**DOI:** 10.3390/plants15131936

**Published:** 2026-06-23

**Authors:** Ayesha Mariam, Chethan Kumar B G, Kalabharathi H L, Nehal Rajendra Stalekar, Benin Yonathan Francis, Anil Kumar K M, Maciej Przybyłek, Ramith Ramu

**Affiliations:** 1 Department of Pharmacology, JSS Medical College, JSS Academy of Higher Education and Research, Mysuru 570015, Karnataka, India; drshamaseerjss@gmail.com (A.M.); hlkalabharathi@jssuni.edu.in (K.H.L.); beniya8437@gmail.com (B.Y.F.); 2 Department of Environmental Sciences, School of Life Sciences, JSS Academy of Higher Education and Research (JSS AHER), Mysuru 570015, Karnataka, India; chethankumarbg@jssuni.edu.in (C.K.B.G.); anilkumarenvi@jssuni.edu.in (A.K.K.M.); 3 Department of Biotechnology and Bioinformatics, School of Life Sciences, JSS Academy of Higher Education and Research (JSS AHER), Mysuru 570015, Karnataka, India; nehal.stalekar19@gmail.com; 4 Department of Physical Chemistry, Faculty of Pharmacy, Collegium Medicum in Bydgoszcz, Nicolaus Copernicus University in Toruń, Kurpińskiego 5, 85-950 Bydgoszcz, Poland

**Keywords:** *Firmiana colorata*, pharmacognosy, ethnopharmacology, phytochemistry, biological activity

## Abstract

*Firmiana colorata* (Roxb.) R.Br. is a medicinal tree used in traditional systems of the Indian subcontinent, but its pharmacognostic profile remains insufficiently consolidated. This PRISMA-guided review integrates the available evidence on its taxonomy, distribution, botanical characteristics, genetic background, phytochemistry, and biological activity, with explicit consideration of nomenclatural synonyms during database searching. The reviewed literature indicates that *F. colorata* contains diverse phenolic, flavonoid, coumarin, lipid, and other specialized metabolites, while pharmacological studies suggest activity mainly in inflammation-, pain-, oxidative stress-, microbial-, and cancer-related experimental models. However, the current evidence remains fragmented and is still dominated by crude extracts, preliminary assays, limited mechanistic validation, and insufficient safety data. Plastome-level information supports taxonomic authentication and evolutionary placement, but the relationship between genetic background and phytochemical variability remains unresolved. The available data therefore justify further investigation of *F. colorata*, provided that future studies emphasize phytochemical standardization, validated mechanisms, dose justification, toxicity assessment, and translational relevance.

## 1. Introduction

Plant-derived natural products remain an important source of structurally diverse molecules for therapeutic discovery [1,2]. Although combinatorial chemistry and high-throughput screening have expanded discovery strategies, natural products retain value because of their chemical diversity, stereochemical complexity, and biosynthetic variation [2]. These features support their use as lead compounds, templates for semisynthetic optimization, and inspiration for medicinal chemistry [1,2]. Genomics-, metabolomics-, and bioinformatics-guided approaches have further improved dereplication and early functional characterization [3].

Within this broader ethnopharmacological setting, the Indian subcontinent is important because of its floristic diversity and long-established medical traditions [4]. Ayurveda, Siddha, and Unani use numerous plant-derived materials, and historically used species can provide rational starting points for phytochemical and pharmacological study [4]. However, translation of traditional medicinal knowledge into broadly accepted applications requires taxonomic authentication, reproducible quality control, phytochemical characterization, and clearer mechanistic interpretation of biological activity [5].

Malvaceae sensu lato, including Sterculiaceae-related taxa and allied genera, provides a relevant reference point because members of this group contain diverse secondary metabolites of pharmacognostic interest [6,7]. Gum-producing members of the *Sterculia* lineage are also relevant to formulation and drug-delivery research [8]. This makes the lineage a useful context for assessing less consolidated taxa such as *F. colorata* [6,7,8].

Ethnobotanical records can help identify species and indications worth further study, but they do not establish efficacy or safety [9]. Traditional-use data may also help prioritize plants for bioprospecting when recurring uses are followed by experimental validation [10]. In this context, *F. colorata* is documented in geographically distinct use records. In Village Common Forests of the Chittagong Hill Tracts, Bangladesh, it is recorded as “Huri”, with bark and root-juice preparations used for hysteria, jaundice, seminal emission, spermaturia, sores, and cholera [11]. In Sylhet district, Bangladesh, its leaf juice was included in a multi-plant oral formulation for intestinal dysfunction used by Oraon and Gor healers [12]. In Maharashtra, the Bhilla tribe used seed powder mixed with wheat flour as laddus for general weakness [13].

These records show that traditional use of *F. colorata* is geographically dispersed and plant-part-specific. However, the correspondence between traditional indications and modern experimental evidence remains incomplete. Pain-, fever-, inflammation-, oxidative-stress-, and antimicrobial-related endpoints have been explored in preliminary pharmacological studies, whereas traditional uses related to cholera, jaundice, intestinal dysfunction, sores, and general weakness remain weakly validated or unvalidated in modern disease-relevant models [14,15,16]. This mismatch between ethnomedicinal breadth and experimental depth provides the rationale for a structured pharmacognostic review focused on taxonomy, historical synonymy, phytochemistry, biological activity, methodological limitations, and safety-oriented standardization.

## 2. Methodology

This review was designed and reported in accordance with PRISMA 2020 [17]. The formal PRISMA-based evidence corpus was restricted to literature directly relevant to *F. colorata*, with an emphasis on ethnobotanical use, pharmacognosy, phytochemistry, analytical characterization, biological activity, and directly informative species-level botanical records.

To ensure a comprehensive retrieval of the literature, the search strategy was designed to rigorously account for the dynamic nature of botanical nomenclature and plant taxonomy. Scientific literature, particularly historical ethnobotanical records, early pharmacognostic reports, and foundational phytochemical studies, frequently employs basionyms or historical synonyms reflecting earlier taxonomic classifications. Searching only for the currently accepted name would have missed critical data. To avoid this, we first established and cross-checked the taxonomic and nomenclatural profile of the species using Plants of the World Online (POWO) [18], the International Plant Names Index (IPNI) [19], and World Flora Online (WFO) [20]. The use of curated nomenclatural and taxonomic resources for plant-name standardization, synonym verification, and taxonomic context is consistent with established practice in botanical research [21,22,23]. Occurrence-data resources such as the Global Biodiversity Information Facility (GBIF) [24] can support distributional interpretation, provided that records are checked for taxonomic consistency, geographic plausibility, and data-quality limitations [25]. This cross-check confirmed the accepted name and the main nomenclatural variants relevant to the search strategy. Accordingly, the primary search strategy for each bibliographic database combined the accepted name, *Firmiana colorata* (Roxb.) R.Br., with its basionym and relevant historically applied synonyms, namely: *Sterculia colorata* Roxb., *Erythropsis colorata* (Roxb.) Burkill, *Erythropsis roxburghiana* Schott & Endl., *Clompanus colorata* (Roxb.) Kuntze, *Karaka colorata* (Roxb.) Raf., *Firmiana rubriflora* Kosterm., and *Sterculia rubicunda* Wall. ex Mast.

This comprehensive taxonomic block was then combined with three thematic concept blocks addressing: (1) taxonomy, distribution, authentication, and genomics; (2) pharmacognosy, phytochemistry, and analytical characterization; and (3) ethnobotany, traditional use, pharmacology, and bioactivity. Across the three platforms, the thematic vocabulary included terms such as taxonom*, nomenclat*, synonym*, authentication, voucher*, morpholog*, distribution, plastome, genome, pharmacognos*, phytochem*, chemotaxonom*, metabol*, extract*, constituent*, flavonoid*, coumarin*, chromatograph*, spectroscop*, HPLC, NMR, ethnobot*, ethnomed*, traditional use, folk medicine, medicinal, pharmacolog*, bioactiv*, antioxidant*, antiinflammat*, analges*, antipyret*, cytotox*, antimicrobial*, toxicity, and safety. No restrictions on publication year, language, or document type were applied at the identification stage. The review workflow began with taxonomic, nomenclatural, and distributional source verification, which informed the subsequent bibliographic searches and screening process. These searches and verifications were conducted in April and May 2026 (last search: 31 May 2026).

Records retrieved from the four query sets within each database were first deduplicated within the source, using database-specific identifiers with supplementary DOI and title checks. This yielded 16 source-unique records from Scopus, 13 from Web of Science Core Collection, and 3 from PubMed, from raw totals of 32, 23, and 9, respectively. The three source-unique sets were then merged and deduplicated across databases by DOI and title matching, with manual harmonization of minor title-format differences, leaving 18 non-duplicate records for integrated screening. Eligibility assessment was conducted at the integrated record level and was based on the available title, abstract, keywords, and full-text content. All integrated records and reports sought for retrieval were screened independently by two reviewers against the predefined inclusion and exclusion criteria. Any differences in opinion were settled by mutual agreement. No automation tools were used to make eligibility decisions. Formal inter-reviewer agreement statistics were not calculated because the final corpus was small and methodologically heterogeneous.

**Figure 1:**
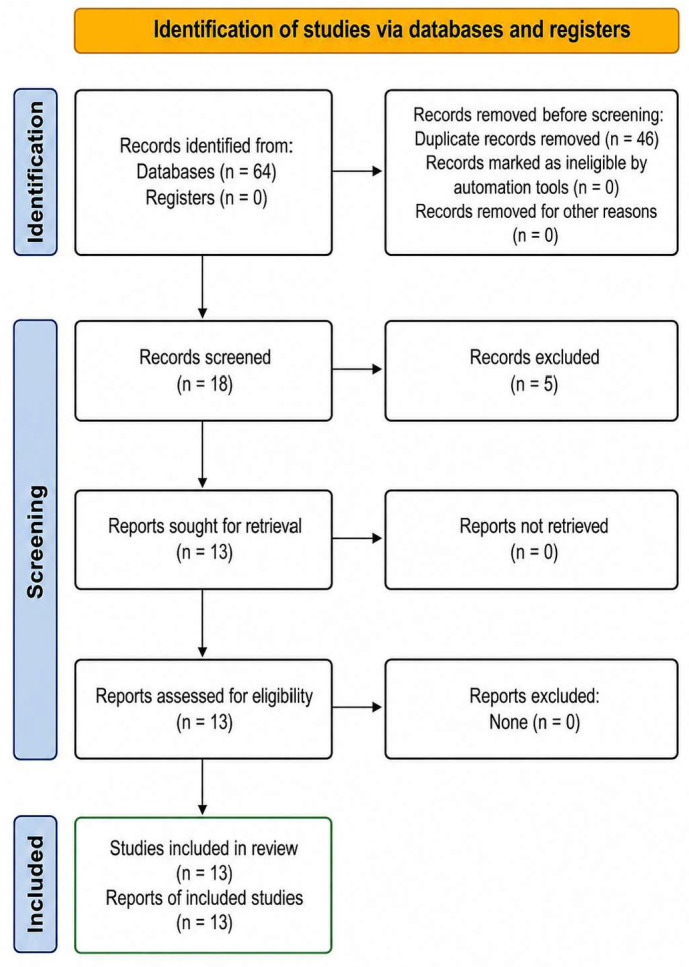
PRISMA 2020 flow diagram adapted for the present review, summarizing identification, deduplication, screening, and final inclusion of bibliographic records [17].

Records were retained in the core PRISMA corpus only when they met the predefined inclusion criteria and addressed *F. colorata* or an accepted synonym directly, or when multispecies or comparative papers contained explicit data attributable to the target species. Records concerning non-target taxa, correction or erratum notices, and records lacking sufficiently direct relevance to the predefined species-specific review scope were excluded. This process resulted in 13 included records. The integrated screening workflow is summarized in Figure 1. To provide a broader context, subsequent sections of this review refer to additional data that fall outside the formal PRISMA methodology.

Consistent with PRISMA 2020 reporting guidance [17], POWO [18], IPNI [19], WFO [20], GBIF [24], and selected regulatory or conservation-status web resources were treated as consulted web-based and organizational resources rather than as primary bibliographic study records. Accordingly, these resources were not counted among the records included in the PRISMA flow diagram. GBIF occurrence data [24] were used only to support the distributional overview. Before interpretive use, these records were checked for taxonomic consistency and geographic plausibility, and they were not used as specimen-level authentication evidence. Data extraction from the included records covered bibliographic information, the specific taxon name used in the original source, study domain, plant part or biological material, extract, fraction, or compound information, analytical approach, study model, and principal findings. Because the final corpus comprised heterogeneous botanical, ethnomedicinal, phytochemical, and pharmacological records, synthesis was conducted as a structured narrative rather than as a quantitative meta-analysis. No formal risk-of-bias tool was applied because the included evidence was methodologically heterogeneous and not amenable to a single validated appraisal instrument.

## 3. Geographic Distribution

*F. colorata*, known across the Indian subcontinent by local names such as “Jari-udal”, “Mula”, and “Udal”, has a broad but habitat-associated reported distribution [16,26]. Historical taxonomic works document the species under *Sterculia colorata* on the Coromandel Coast [19], while its later placement within *Firmiana* situates it in a broader South and Southeast Asian floristic context [19,20]. In India, the species is reported from the Konkan region and the Deccan plateau [14,18], the moist deciduous forests of South Dangs [26], lowland forest reserves in Assam [27], and the southern Indian Peninsula, including the Pondicherry area [16].

Bangladesh is also an important occurrence and traditional-use area for *F. colorata*. Recent pharmacological collections, ethnobotanical surveys, and taxonomic records document the species from the Chattogram area/division, the Chittagong Hill Tracts, Rangamati, Sylhet, and other districts, including herbarium records from 1947 to 2022 [11,15,16,28]. In the Chittagong Hill Tracts, *F. colorata* is recorded from Village Common Forests [11], and ethnobotanical sources report its local name “Huri” and use by Oraon and Gor healers in Sylhet district [11,12]. These records support its ethnobotanical relevance, but they remain contextual evidence of use rather than proof of pharmacological efficacy.

**Figure 2:**
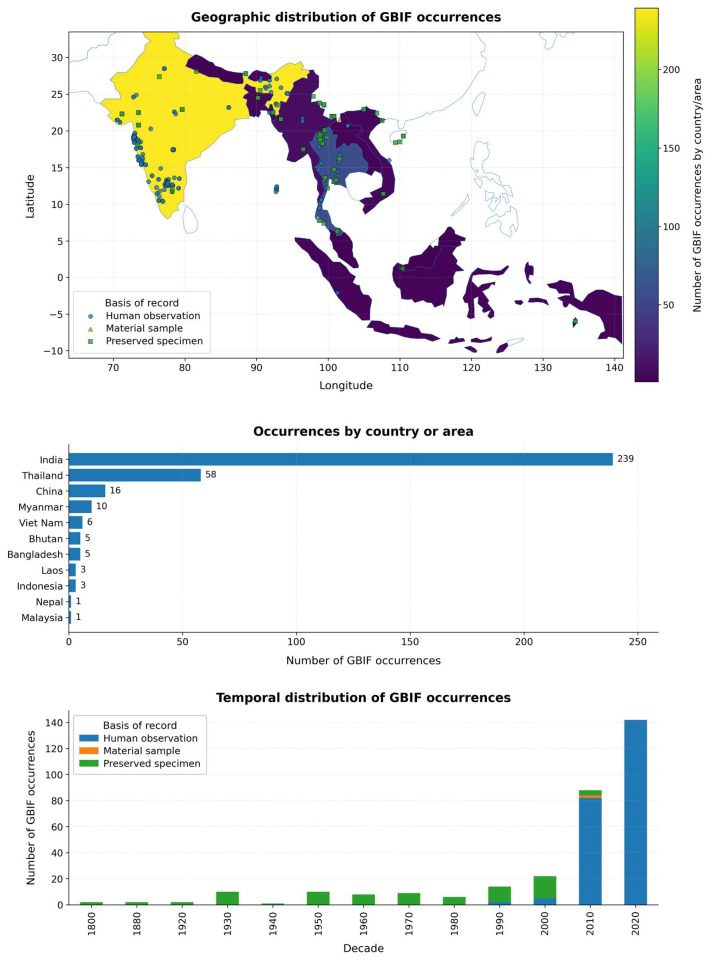
Geographic and temporal overview of *Firmiana colorata* occurrence records from the Global Biodiversity Information Facility (GBIF) [24] across South and Southeast Asia.

*F. colorata* is a tropical deciduous tree associated with seasonal habitat conditions [18,20]. Its phenology responds to dry and wet seasonal contrasts, including leaf shedding during cooler dry periods and renewed vegetation during humid monsoon conditions [29]. This is consistent with reported occurrences in seasonally dry subtropical forests across the Indian subcontinent, Myanmar, the Indochinese Peninsula, and southern China [14,18]. The approximate native distribution according to GBIF [24] is summarized in Figure 2. Globally, *F. colorata* is listed as Least Concern in World Flora Online resources linked to the International Union for Conservation of Nature (IUCN) [20], while POWO reports its native range from the Indian subcontinent to southern Yunnan and Sumatera [18]. Regional pressure may still occur, as the Plants Red List of Bangladesh classifies the species as Near Threatened [30].

As shown in Figure 2, GBIF records [24] are concentrated mainly in India, with additional records from Thailand, China, Myanmar, Vietnam, Bangladesh, and neighboring areas. The dataset combines historical specimen-based documentation with recent observation-based biodiversity reporting and should therefore be interpreted as a distributional overview rather than as specimen-level authentication evidence.

## 4. Botanical Characteristics

### 4.1. Macromorphological Diagnostic Features

*F. colorata* is macroscopically characterized as a medium-sized to large deciduous tree, capable of reaching heights of up to 15 m, with a spreading branching architecture [20,29]. Floristic descriptions characterize the trunk as ash-colored, dried young branchlets as gray-black and finely gray-puberulent, and leaves as broadly cordate, 17.5–25 cm long and up to 18 cm wide, with 10–15 cm petioles and crowding near the branch tips [20,31].

### 4.2. Wood Anatomical Features

Anatomically, *F. colorata* features a storied vascular cambium with compound heterocellular rays and sheath cells. Its structure includes diffuse-porous secondary xylem and secondary phloem composed of sieve tube members, companion cells, parenchyma, and fibers [32].

### 4.3. Phenology and Reproductive Features

**Figure 3:**
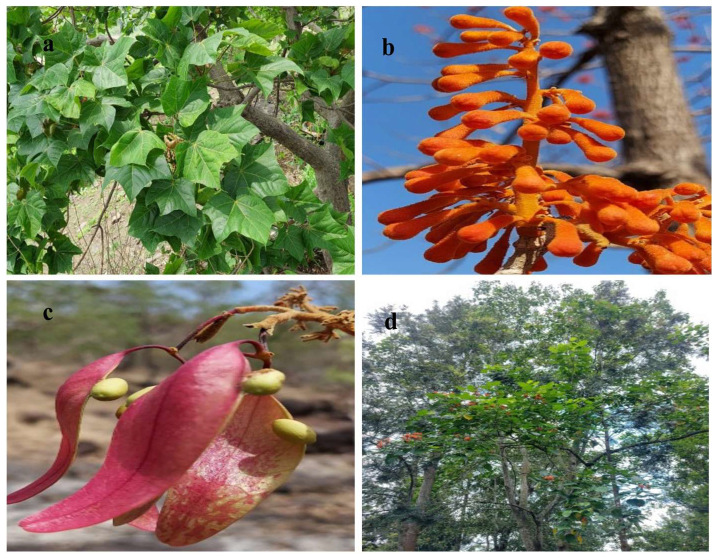
Representative vegetative and reproductive morphology of *Firmiana colorata*. (**a**) Leafy branchlet with broadly cordate, palmately veined leaves. (**b**) Flowering inflorescence bearing orange-red pubescent flowers and flower buds. (**c**) Fruiting structure composed of open follicles with exposed seeds. (**d**) Whole-tree habit with a spreading crown.

*F. colorata* is associated with wet tropical to seasonally deciduous forest habitats, where moisture seasonality supports its deciduous habit [18,20]. In a tropical moist-forest study from the Indo-Burma hotspot, the species flowered during a complete leafless period, consistent with floristic descriptions of prominent flowers emerging before new leaves on bare branches [29,31]. This leafless flowering pattern may improve floral visibility and remains relevant to species-level recognition [27,29]. Representative morphological features are shown in Figure 3.

## 5. Genetic Aspects

Genetic information is relevant to medicinal-plant research because it supports taxonomic authentication and helps define the biosynthetic background of plant material. For *F. colorata*, current data do not yet establish a direct relationship between environmental conditions and phytochemical variability. Evidence from broader medicinal-plant research indicates that light exposure, temperature, water availability, soil properties, provenance, and season can influence secondary-metabolite accumulation [33,34,35]. These factors should guide future standardization of *F. colorata*, particularly because genetically authenticated material may still require chemical verification across habitats or seasons.

To date, only one comprehensive study has sequenced and characterized the complete plastid genome of *F. colorata* [36]. According to this report, the complete plastome is a 160,700 bp circular molecule with the typical angiosperm quadripartite structure, comprising an 89,551 bp LSC region, a 20,001 bp SSC region, and two 25,574 bp inverted repeats, with an overall GC content of 37.1%. The plastid genome contains 130 genes, including 113 distinct genes, and shows intron-containing loci, trans-splicing of rps12, selected gene overlaps, and A/T-biased codon usage at the third position. RNA-editing predictions identified 65 sites across 24 genes, mainly involving C-to-U conversion and affecting several chloroplast transcripts. The plastome also contains abundant simple sequence repeats and larger repeats, while most protein-coding genes appear to be under purifying selection. In contrast, rps4, accD, and rpl20 showed dN/dS ratios greater than 1, suggesting possible positive selection without established functional consequences for metabolite variation in *F. colorata* [36].

Although direct gene-to-metabolite relationships have not been demonstrated in *F. colorata*, the plastome data allow cautious connections with its phytochemical profile. The most defensible lipid-related link concerns accD, which encodes a subunit of acetyl-CoA carboxylase and is therefore related to upstream fatty-acid precursor metabolism [37,38]. This should not be read as direct evidence for control of cyclopropenoid fatty acid formation, because these compounds are generated later by dedicated synthases acting on unsaturated substrates [39,40]. A second link concerns chloroplast function, since RNA editing supports chloroplast protein accumulation and development, while chloroplast status can influence nuclear responses through retrograde signaling [41,42,43]. This provides a plausible basis for connecting plastome-regulated chloroplast performance with the metabolic conditions that support phenolic and flavonoid accumulation, because flavonoid biosynthesis can respond to photosynthetic redox status [44,45]. The antioxidant-related phytochemical profile also fits chloroplast biochemistry, since homogentisate, phytol, and the plastidial MEP pathway are linked to tocopherols, chlorophyll side chains, phylloquinol/plastoquinone-related metabolism, and prenylquinones [46,47,48,49]. The repeat-rich plastome is therefore best viewed as a future tool for authentication and population-level comparison rather than as a direct determinant of metabolite biosynthesis [50].

## 6. Phytochemical Architecture

Assessment of the pharmacognostic potential of *F. colorata* requires a clear synthesis of its reported phytochemical profile. Available studies show that the species contains diverse specialized metabolites and lipophilic constituents across different plant organs (Table 1). This phytochemical spectrum is relevant for bioactivity assessment, as flavonoid- and phenolic-related constituents support antioxidant-oriented hypotheses, whereas seed cyclopropenoid fatty acids introduce an important safety dimension. In pharmacognosy, the biological relevance of such compounds is structure-, exposure-, and model-dependent, encompassing both potentially beneficial activities and adverse or toxicological effects [51].

The earliest focused phytochemical study of *F. colorata* leaves identified a flavonoid-rich polar fraction and reported a C-6 glucuronylated, 6-oxygenated flavone together with additional flavonoid-related constituents [52]. Beyond expanding the constituent list, this finding is important because it anchors the leaf chemistry of *F. colorata* in oxygenated flavone derivatives with pharmacognostic and potential chemotaxonomic relevance.

Further phytochemical work extended the chemical profile of *F. colorata* by reporting two coumarin derivatives from the ethyl acetate fraction of *Sterculia colorata*: 4-(3,5-dihydroxyphenyl)-3,5,7-trihydroxy-2H-chromen-2-one and 4-(3,4-dihydroxyphenyl)-5,7-dihydroxy-3-(((2R,3S,4R,5R,6S)-3,4,5-trihydroxy-6-(hydroxymethyl)tetrahydro-2H-pyran-2-yl)oxy)-2H-chromen-2-one. These compounds were reported as new by the original authors and were structurally characterized using MS, UV, IR, ^1^H NMR, ^13^C NMR, HSQC, HMBC, and COSY analyses. The HMBC correlations supported the assignment of the substituted aromatic moieties and confirmed the proposed coumarin frameworks [26]. These structural data are chemically informative, although the contribution of individual coumarins to the biological activity of crude extracts still requires direct compound–activity correlation.

Qualitative screening of bark and leaf methanolic extracts, together with GC-MS profiling of bark extract, further broadened the known chemical profile of the species. These studies reported alkaloids, phenolic compounds, tannins, glycosides, saponins, fatty acid esters, phenolic acids, and triterpene-related constituents, with saponins reported in bark but absent from the leaf extract. These findings are useful for mapping chemical diversity, but most of them remain qualitative or profile-based rather than quantitatively standardized by marker compounds [15,16].

The seed oil represents a distinct non-polar phytochemical compartment. Lipid analysis identified cyclopropenoid fatty acids, including malvalic and sterculic acids, together with oleic, palmitic, linoleic, and related fatty acids. This seed profile complements the polar flavonoid and coumarin evidence from vegetative tissues and confirms that *F. colorata* contains chemically divergent metabolite pools across plant organs [31]. Key spectroscopic and chromatographic markers supporting the structural characterization of *F. colorata* constituents are summarized in Table 2.

**Figure 4:**
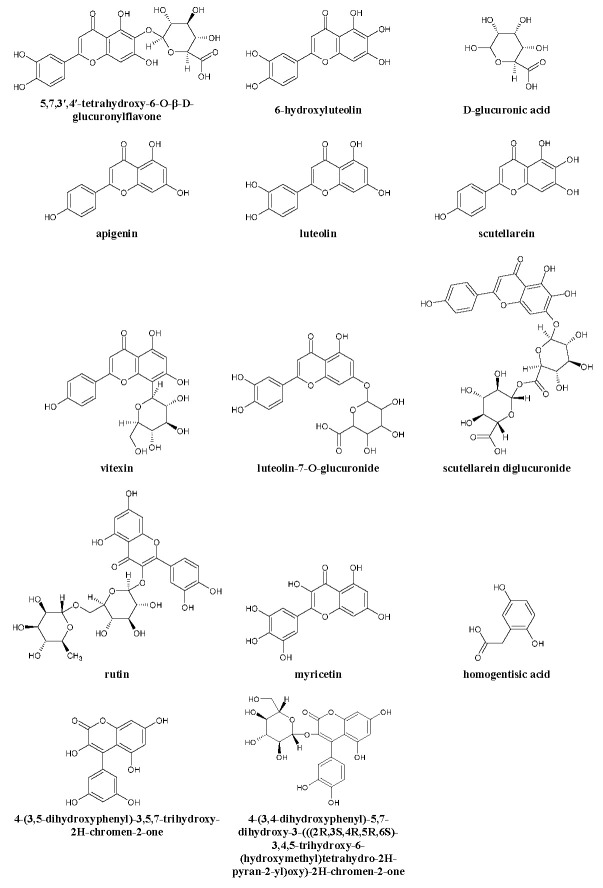
Chemical structures of reported flavonoid-related, coumarin-related, and phenolic constituents, as well as hydrolysis products.

**Figure 5:**
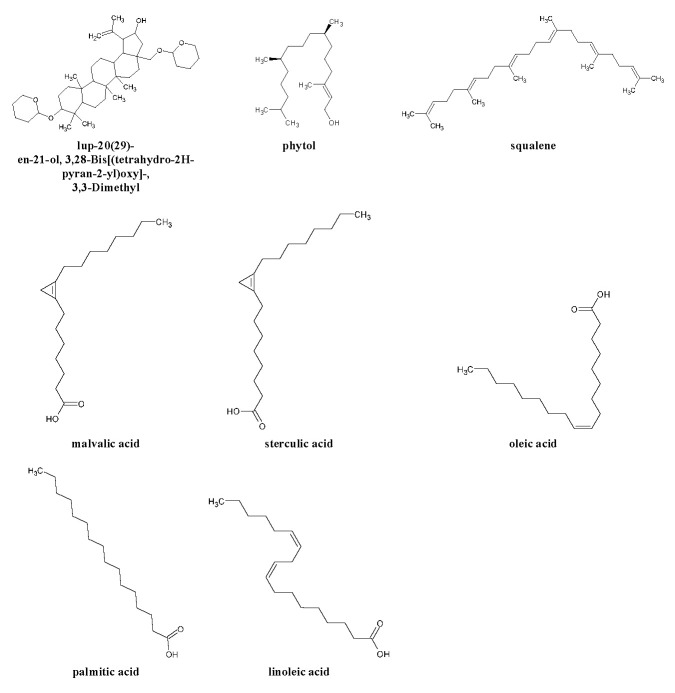
Chemical structures of reported terpenoid/lipophilic constituents and fatty acids.

The chemical structures compiled in Figure 4 and Figure 5 correspond to the main constituent groups discussed above, including flavonoid-related, coumarin-related, and phenolic compounds, hydrolysis products, terpenoid/lipophilic constituents, and fatty acids.

## 7. General Pharmacology

**Figure 6:**
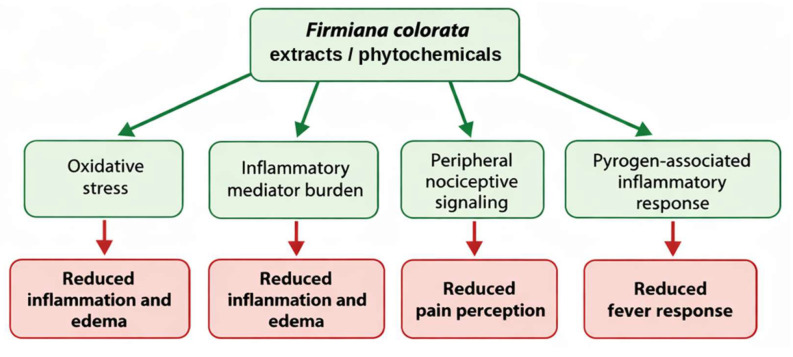
Conceptual overview of the principal anti-inflammatory, antinociceptive, and antipyretic response domains associated with *Firmiana colorata* extracts and phytochemicals based on the collected sources [14,15,16].

**Figure 7:**
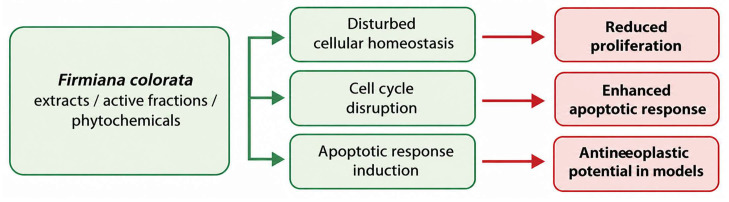
Conceptual overview of the principal antineoplastic response framework associated with *Firmiana colorata* extracts, active fractions, and phytochemicals based on the collected sources [26,57].

The reported pharmacological profile of *F. colorata* should be read cautiously because botanical extracts are chemically complex mixtures [53,54]. Additivity or synergy cannot be inferred from complexity alone and requires dedicated experimental confirmation [55,56]. Figure 6 therefore summarizes the main reported anti-inflammatory, antinociceptive, and antipyretic activity areas as a conceptual overview rather than a validated molecular network. Figure 7 and Table 3 further synthesize the antineoplastic findings and the broader activity profile.

The major pharmacological domains of *F. colorata* are best interpreted as partially interconnected response patterns rather than fully separate bioactivities. The available evidence for antiproliferative activity points particularly to disturbed cellular homeostasis, cell-cycle disruption, and apoptosis induction, as outlined in Figure 7. Table 3 summarizes the pharmacological activities reported for *F. colorata*, including the evaluated extracts or compounds, experimental models, and key observations discussed in the following subsections.

### 7.1. Analgesic, Anti-Inflammatory, and Antipyretic Activities

Recent preclinical studies indicate that *F. colorata* has antinociceptive, anti-inflammatory, and antipyretic activity in murine models, with additional numerical details provided in Table 3. The available evidence is based mainly on a methanolic bark extract (MEFCB) and a highly lipophilic n-hexane leaf fraction (NHFC) evaluated in classical nociception, inflammation, and pyrexia assays [15,16]. The acetic acid-induced writhing test models chemically induced visceral nociception involving algogenic and pro-inflammatory mediators, including prostaglandins and bradykinin [58,59]. The formalin test complements this model by separating an early neurogenic phase, associated mainly with peripheral nociceptor activation, from a late phase involving inflammatory signaling and sensitization-related mechanisms [60,61,62].

Within this framework, MEFCB produced a dose-dependent reduction in writhing responses, increasing from 23.52% inhibition at 200 mg/kg to 35.33% at 400 mg/kg [16]. NHFC showed stronger inhibition in the same type of chemically induced pain model, reaching 81.78% at 400 mg/kg, and also produced marked activity in the late inflammatory phase of the formalin test [15]. These findings support peripheral and inflammation-related antinociceptive activity, although direct pharmacological equivalence with diclofenac sodium cannot be claimed because the extract and the reference drug differ in dosage, composition, and standardization [15,16].

MEFCB also attenuated carrageenan-induced paw edema, supporting an in vivo anti-inflammatory effect at the phenotypic level [16]. Docking results suggested possible interactions of selected constituents with COX-1 and COX-2, but this remains a mechanistic lead rather than biochemical confirmation because PGE_2_ production, COX-2 expression or activity, IL-6, and other inflammatory cytokines were not measured [16].

Both preparations also reduced yeast-induced fever at 400 mg/kg, with maximum reported reductions of 92.52% for MEFCB and 88.96% for NHFC, numerically close to paracetamol in the same assay [15,16]. This antipyretic profile is chemically plausible in view of the reported flavonoid- and tannin-related constituents [15,16], and is consistent with broader evidence that plant-derived polyphenols can modulate fever-related inflammatory pathways, including cyclooxygenase-dependent prostaglandin E_2_ formation and endogenous pyrogen release [63,64,65].

### 7.2. Immunomodulatory and Antioxidant Effects

Oxidative stress contributes to the progression of many chronic inflammatory disorders [66,67], but the reported antioxidant activity of *F. colorata* extracts should be interpreted as screening-level evidence. This activity is chemically consistent with the presence of phenolic, flavonoid-related, and lipophilic constituents, including homogentisic acid, rutin, myricetin, phytol, and squalene [14,16,26]. However, the available studies do not quantify the individual contribution of these compounds, because LC-MS-based quantification and compound–activity correlation analyses have not been reported. As noted in Table 3, the current antioxidant evidence is based mainly on in vitro radical-scavenging and metal-chelating assays [14,15]. The dichloromethane fraction showed DPPH radical neutralization, other fractions scavenged hydrogen peroxide [15], and aqueous leaf extracts showed NO radical-scavenging and iron-chelating activities [14]. Because excessive NO can contribute to peroxynitrite formation and oxidative-inflammatory injury, these assays provide relevant screening evidence but do not establish in vivo antioxidant efficacy [68,69,70]. Isolated coumarin derivatives also showed immunomodulatory activity in luminol-enhanced chemiluminescence assays, with IC_50_ values in the same experimental range as ibuprofen [26]. Docking results suggested possible interactions of lipophilic constituents with FOXO- and NF-κB-related targets [14], but these findings remain hypothesis-generating and do not show direct regulation of antioxidant or inflammatory pathways in vivo.

### 7.3. Antineoplastic, Pro-Apoptotic, and Cell Cycle Regulatory Activity

As detailed in Table 1 and the antineoplastic section of Table 3, studies on *Sterculia colorata*, a synonym of *F. colorata*, led to the isolation of two coumarin derivatives reported by the original authors as new compounds, and these were later evaluated in HL-60 leukemia cells [26,57]. Molecular docking suggested favorable interactions with CDK-2, and MTT assays showed significant cytotoxicity, supporting a hypothesis of cell-cycle perturbation consistent with G1-phase arrest [57]. This interpretation is biologically plausible because CDK-2 regulates the late G1/S transition through cyclin E-dependent Rb phosphorylation and E2F-mediated S-phase entry [71,72,73,74,75,76,77]. Docking also suggested possible interaction with BCL-2 [57], a regulator of the intrinsic mitochondrial apoptotic pathway involving Bax/Bak-dependent pore formation, cytochrome c release, and caspase activation [78,79]. These findings suggest possible involvement of cell-cycle regulation and intrinsic apoptosis, but this interpretation still requires direct experimental confirmation [57].

### 7.4. Antimicrobial Properties of Extracts and Biosynthesized Silver Nanoparticles

The phytochemical profile summarized in Table 1 provides a reasonable basis for antimicrobial screening because *F. colorata* contains flavonoid- and phenolic-related constituents, tannins, saponins, and triterpene-related metabolites, which are frequently discussed among plant-derived antimicrobial agents [80,81]. Species-specific evidence currently derives from one study of aqueous leaf extract and corresponding biosynthesized AgNPs [14]. Using agar well diffusion, the study reported inhibition zones against Staphylococcus aureus, *Bacillus subtilis*, *Escherichia coli*, and *Klebsiella* sp. Both preparations were active under the applied conditions, and the AgNP preparation produced larger zones than the crude extract. These data indicate antibacterial potential and the ability of the extract to mediate AgNP formation, but they remain descriptive because MIC/MBC values, dose-normalized comparisons, and statistical confirmation were not reported.

In the same study, AgNPs were characterized by UV–Vis spectroscopy, FTIR, SEM, and EDX, with SEM indicating mostly spherical particles of approximately 60–90 nm and some irregular particles. FTIR and EDX supported the involvement of extract-derived biomolecules in nanoparticle formation and surface association, linking *F. colorata* phytochemistry with green nanomaterial synthesis. Antibacterial and antifungal activity has also been reported for the related species *Firmiana simplex* (L.) W.Wight, where leaf and bark extracts were evaluated using inhibition-zone and MIC assays against selected bacterial and fungal strains [82]. This genus-level comparison supports further comparative screening of *Firmiana* taxa, but it should not be treated as direct evidence for *F. colorata* potency or mechanism.

### 7.5. Evidence Status, Standardization, and Translational Readiness

The studies summarized above support a credible pharmacological research profile for *F. colorata*, but they also show that the literature remains at an early translational stage. The bark-extract study is methodologically informative because it included plant authentication, vehicle and positive-control groups, acute oral toxicity assessment, LD_50_-based dose selection, *n* = 5 animals per group in the in vivo assays, and one-way ANOVA followed by Dunnett’s test [16]. The leaf-extract study similarly included vehicle and standard-drug groups, *n* = 5 animals per group, and the same statistical approach [15]. These features strengthen the initial evidence for analgesic, antipyretic, anti-inflammatory, and antioxidant activity. Nevertheless, the available literature still lacks broader studies that integrate dose justification, quantitative marker-based standardization, direct measurements of inflammatory mediators, COX/LOX activity, pathway-specific biomarkers, and in vivo antioxidant endpoints within the same experimental framework.

The isolated-coumarin study provides a chemically more defined contribution because individual compounds were evaluated in immunomodulatory and PC-3 anticancer assays with reference drugs and triplicate measurements [26]. At present, however, the literature does not yet provide a wider compound-level evaluation across normal-cell selectivity models, broader cancer-cell panels, and downstream biomarkers of cell-cycle regulation, apoptosis, immune modulation, and oxidative stress. Such studies would help connect isolated constituents with the activity patterns described for crude extracts and fractions.

The antimicrobial and AgNP study expands the research profile by linking leaf-extract chemistry with biosynthesized nanomaterials characterized by UV–Vis spectroscopy, FTIR, SEM, and EDX [14]. Because the available species-specific antimicrobial data are based mainly on agar well diffusion and inhibition-zone measurements, the literature still lacks MIC/MBC values, dose-normalized comparisons, and statistically supported analyses of antibacterial potency. Further work should also broaden nanomaterial characterization by including DLS-based size distribution, polydispersity index, zeta potential, and storage stability. Control designs that distinguish extract-derived effects, particle-associated effects, and combined or potentially synergistic responses would be especially useful for interpreting the contribution of phytochemicals to AgNP-mediated activity. Safety-oriented assays, including mammalian-cell cytotoxicity, hemocompatibility, and in vivo toxicity assessment, would further strengthen translational interpretation.

From a regulatory and translational perspective, no specific GRAS notice or food-use GRAS determination was identified for *F. colorata* or its extracts. GRAS refers to safety under specified food-use conditions, not to therapeutic efficacy or approved medicinal dosing [83]. Therefore, traditional use, preliminary pharmacological activity, or the presence of individual metabolites should not be interpreted as regulatory acceptance of *F. colorata* preparations for food, nutraceutical, or therapeutic use. FDA GRAS entries for chemically related substances or broad ingredient categories, such as tocopherols, phytosterols, and fatty-acid-rich oils, refer to defined substances, sources, specifications, and intended food-use conditions, and do not establish GRAS status for *F. colorata* or its extracts. No approved human therapeutic dosage was identified. Future translational development would require standardized extract characterization, batch-to-batch quality control, justified dosing, targeted toxicological evaluation, and safety-oriented quality control.

## 8. Conclusions

*F. colorata* emerges from the present review as a medicinal plant of clear pharmacognostic interest, although its therapeutic profile remains insufficiently defined. The available literature links its ethnomedicinal relevance with a chemically diverse metabolite profile and several preliminary biological effects. Reported constituents include flavonoids, coumarins, phenolic compounds, terpenoid-related metabolites, and distinctive lipid components, while pharmacological studies indicate analgesic, anti-inflammatory, antipyretic, antioxidant, immunomodulatory, antimicrobial, and antineoplastic potential. These findings support further pharmacognostic investigation of *F. colorata*, but they do not establish it as a clinically validated medicinal species.

The evidence base remains fragmented. Much of the available work relies on crude extracts, fractions, preliminary in vitro assays, animal models, or docking-supported interpretations. Compound-to-activity relationships, quantitative marker-based standardization, dose justification, mechanistic validation, and safety-oriented evidence remain limited. Plastome-level data support taxonomic and evolutionary interpretation, but they do not yet explain how genetic background, plant organ, provenance, or environmental conditions shape the phytochemical and biological profile of the species.

Future research should therefore move from preliminary characterization toward more integrated pharmacognostic evaluation. Priority should be given to organ- and provenance-resolved metabolite profiling, broader analytical approaches, bioassay-guided isolation of active constituents, and reproducible quantitative standardization of extracts. These studies should be combined with mechanism-oriented assays, toxicity and safety evaluation, pharmacokinetic characterization, and stronger in vivo validation. Such work would clarify whether *F. colorata* can support lead-compound discovery or standardized botanical development. At present, it remains a promising but insufficiently resolved pharmacognostic resource.

## Figures and Tables

**Table 1:** Reported Phytochemical Constituents and Metabolite Classes of *Firmiana colorata*.

Phytochemical Class	Specific Identified Constituents and Related Findings	Plant Part	Extraction/Fractionation	Analytical/Identification Method	Ref.
Flavonoids and glycosides	5,7,3′,4′-tetrahydroxy-6-*O*-β-D-glucuronylflavone; 6-hydroxyluteolin and D-glucuronic acid as hydrolysis products; additional flavonoid-related constituents reported after hydrolysis, including apigenin, luteolin, scutellarein, 6-hydroxyluteolin, vitexin, luteolin-7-*O*-glucuronide, and probable scutellarein diglucuronide	Leaves	Hot ethanol extraction followed by sequential solvent fractionation with solvents of increasing polarity, including benzene, ether, ethyl acetate, methyl ethyl ketone, and the aqueous mother liquor	UV spectroscopy, color reactions with NH_3_ and Fe^3+^, acid hydrolysis, β-glucuronidase hydrolysis, paper chromatography, and co-paper chromatography	[52]
Coumarins	4-(3,5-dihydroxyphenyl)-3,5,7-trihydroxy-2H-chromen-2-one and 4-(3,4-dihydroxyphenyl)-5,7-dihydroxy-3-(((2R,3S,4R,5R,6S)-3,4,5-trihydroxy-6-(hydroxymethyl)tetrahydro-2H-pyran-2-yl)oxy)-2H-chromen-2-one	Whole plant	Extraction of shade-dried plant material with 90% methanol, followed by solvent partitioning and isolation from the ethyl acetate fraction using silica-gel column chromatography and TLC-guided subfractionation	MS, UV, IR, ^1^H NMR, ^13^C NMR, HSQC, HMBC, COSY, and DEPT analyses	[26]
Mixed metabolites	Alkaloids, tannins, glycosides, phenols, and saponins, with saponins reported in bark and absent in leaves	Bark and leaves	Methanol extraction	Qualitative colorimetric phytochemical screening	[15,16]
GC-MS-profiled metabolites	Fatty acid esters, phenolic acids, including homogentisic acid, and triterpene-related constituents, including lup-20(29)-en-21-ol, 3,28-Bis[(tetrahydro-2H-pyran-2-yl)oxy]-, 3,3-Dimethyl	Bark	Methanol extraction	Gas chromatography–mass spectrometry	[16]
Lipids and fixed oils	Cyclopropenoid fatty acids, including malvalic and sterculic acids, together with oleic, palmitic, and linoleic acids	Seeds	Non-polar solvent extraction	HBr titration and gas–liquid chromatography	[31]

**Table 2:** Spectroscopic and Chromatographic Markers for *F. colorata* Phytochemicals.

Analytical Technique	Principal Observations/Spectral Values	Structural and Chemical Significance	Ref.
UV Spectroscopy	UV absorption maxima at 259, 270, and 352 nm, with a shoulder at 220 nm; for the aglycone, characteristic absorption band was shifted by 15 nm toward shorter wavelengths.	Indicates the specific flavonoid backbone configuration and glycosylation at C-6.	[52]
Colorimetric Reactions	Bright yellow with NH_3_; Olive green with Fe^3+^.	Chemically confirms the abundant presence of free phenolic hydroxyl groups.	[52]
Acid Hydrolysis (25% HCl under reflux for 3 h)	Cleavage into D-glucuronic acid and 6-hydroxyluteolin.	Confirms the exact O-beta-D-glucuronyl structural linkage.	[52]
2D NMR (HMBC)	Strong correlations: H-6 to C-5, C-7, C-8, C-10; and H-8 to C-7, C-9.	Defines the precise molecular positioning of the 5,7-dihydroxyphenyl moiety.	[26]
GC-MS Profiling	Detection of diverse lipophilic components (e.g., fatty acid esters, phenolic acids, specific triterpenes).	Confirms the presence of a structurally diverse lipophilic matrix in the bark, notably distinct from classical volatile essential oils.	[16]

**Table 3:** Pharmacological activities reported for *Firmiana colorata*.

Pharmacological Activity	Extract Type/Bioactive Constituents	Plant Part	Experimental Model	Key Observations and Mechanistic Insights	Ref.
Analgesic/Antinociceptive	Methanol (MEFCB) and n-Hexane (NHFC) extracts	Bark, Leaves	Acetic acid writhing and Formalin tests (mice)	Dose-dependent reduction in nociceptive behavior in acetic acid-induced writhing and formalin paw-licking tests. MEFCB inhibited writhing by 23.52% at 200 mg/kg and 35.33% at 400 mg/kg, while NHFC reached 81.78% inhibition at 400 mg/kg. In the formalin test, late-phase inhibition reached 75.30% for NHFC and 28.30% for MEFCB. Direct mediator measurements, including PGE_2_, were not reported.	[15,16]
Anti-inflammatory	Methanol extract (MEFCB)	Bark	Carrageenan-induced paw edema (mice)	Significant temporal attenuation of carrageenan-induced paw edema; docking results suggested possible interactions of selected constituents with COX-1/COX-2, but direct measurements of PGE_2_, COX-2 expression/activity, IL-6, cytokines, or COX/LOX enzyme inhibition were not reported.	[16]
Antipyretic	Methanol (MEFCB) and n-Hexane (NHFC) extracts	Bark, Leaves	Brewer’s yeast-induced pyrexia (mice)	Reduced yeast-induced fever, with the strongest effects reported at 400 mg/kg (MEFCB: 92.52%; NHFC: 88.96%). These values approached the paracetamol response in the respective assays, but the mechanism of the antipyretic effect was not directly tested.	[15,16]
Antioxidant	Phenolic acids, flavonoids, phytol, squalene	Leaves	Free radical scavenging assays	In vitro DPPH, H_2_O_2_, NO radical-scavenging and iron-chelating activities were reported; the contribution of individual compounds and FOXO/NF-κB-related mechanisms remains unconfirmed without quantitative profiling and experimental pathway validation.	[14,15]
Antineoplastic	Coumarin derivatives reported as new compounds	Plant material	HL-60 human leukemia cell line	Showed cytotoxic activity in HL-60 leukemia cells. Docking indicated interactions with CDK-2 and BCL-2, but direct evidence of cell-cycle arrest or apoptosis induction was not reported.	[26,57]
Antibacterial/AgNP-associated antimicrobial screening	Aqueous leaf extract and biosynthesized AgNPs	Leaves	Agar well diffusion/inhibition-zone assays against *Staphylococcus* aureus, *Bacillus subtilis*, *Escherichia coli*, and *Klebsiella* sp.	Both the crude extract and AgNP preparation produced inhibition zones under the applied assay conditions, with larger zones reported for the AgNPs. MIC/MBC values, dose-normalized comparisons, formal extract-versus-AgNP statistical comparisons, and separation of extract-derived and particle-associated effects were not reported.	[14]

## Data Availability

The original contributions presented in this study are included in the article/Supplementary Material. Further inquiries can be directed to the corresponding authors.

## Supplementary Materials

The following supporting information can be downloaded at https://www.mdpi.com/article/10.3390/plants15131936/s1, PRISMA 2020 Checklist: completed PRISMA 2020 checklist indicating where each checklist item is reported in the manuscript.
